# The influence of a formula supplemented with dairy lipids and plant oils on the erythrocyte membrane omega-3 fatty acid profile in healthy full-term infants: a double-blind randomized controlled trial

**DOI:** 10.1186/1471-2431-12-164

**Published:** 2012-10-17

**Authors:** Maria Lorella Giannì, Paola Roggero, Charlotte Baudry, Amandine Ligneul, Daniela Morniroli, Francesca Garbarino, Pascale le Ruyet, Fabio Mosca

**Affiliations:** 1 Neonatal Intensive Care Unit (NICU), Department of Clinical Science and Comunity Health, Fondazione IRCCS “Ca’ Granda” Ospedale Maggiore Policlinico, University of Milan, Milan, Italy; 2 Lactalis Recherche et Développement, 8 Fromy - CS 60082, 35240, Retiers, France

**Keywords:** Full-term infants, Formula supplementation, Dairy lipids, Erythrocyte membrane omega-3 fatty acid profile

## Abstract

**Background:**

Human milk is the optimal nutrition for infants. When breastfeeding is not possible, supplementation of infant formula with long chain polyunsaturated fatty acids appears to promote neurodevelopmental outcome and visual function. Plant oils, that are the only source of fat in most of infant formulas, do not contain specific fatty acids that are present in human and cow milk and do not encounter milk fat triglyceride structure. Experimental data suggest that a mix of dairy lipids and plant oils can potentiate endogenous synthesis of n-3 long chain polyunsaturated fatty acids. This trial aims to determine the effect of an infant formula supplemented with a mixture of dairy lipids and plant oils on the erythrocyte membrane omega-3 fatty acid profile in full-term infants (primary outcome). Erythrocyte membrane long chain polyunsaturated fatty acids and fatty acids content, the plasma lipid profile and the insulin-growth factor 1 level, the gastrointestinal tolerance, the changes throughout the study in blood fatty acids content, in growth and body composition are evaluated as secondary outcomes.

**Methods/Design:**

In a double-blind controlled randomized trial, 75 healthy full-term infants are randomly allocated to receive for four months a formula supplemented with a mixture of dairy lipids and plant oils or a formula containing only plant oils or a formula containing plant oils supplemented with arachidonic acid and docosahexaenoic acid. Twenty-five breast-fed infants constitute the reference group. Erythrocyte membrane omega-3 fatty acid profile, long chain polyunsaturated fatty acids and the other fatty acids content, the plasma lipid profile and the insulin-growth factor 1 level are measured after four months of intervention. Gastrointestinal tolerance, the changes in blood fatty acids content, in growth and body composition, assessed by means of an air displacement plethysmography system, are also evaluated throughout the study.

**Discussion:**

The achievement of an appropriate long chain polyunsaturated fatty acids status represents an important goal in neonatal nutrition. Gaining further insight in the effects of the supplementation of a formula with dairy lipids and plant oils in healthy full-term infants could help to produce a formula whose fat content, composition and structure is more similar to human milk.

**Trial registration:**

ClinicalTrials.gov Identifier NCT01611649

## Background

Human milk is recognized as the gold standard for infant nutrition [1-3]. Lipids are a major component of human milk and provide 45% of total energy intake. The main constituents are triacylglycerols, phospholipids and their components fatty acids and sterols. Lipids contribute to several biological functions with regard to growth and development. In particular, human milk provides essential fatty acids (EFA) and long chain polyunsaturated fatty acids (LC-PUFA) of omega-3 and omega-6 families, such as docosahexaenoic acid (DHA, 22:6n-3) and arachidonic acid (AA, 20:4n-6) [4]. These fatty acids constitute the main components of brain tissue and have an important impact on neuronal and visual functions [5]. LC-PUFA accretion in human brain is a slow gradual process [6]. The last trimester of pregnancy represents a critical period for LC-PUFA deposition whose accretion in the nervous system endures after birth [7-9]. The deposit of AA during the last trimester of pregnancy differs from that of DHA so that at term human brain contains relatively more AA than DHA. However, after term, DHA brain content increases, resulting in DHA being the main LC-PUFA in adult brain. It has been demonstrated that lipids represent 50 to 60% of dry adult brain weight [10]. In addition, cerebral LC-PUFA concentration seems to be higher in the cortical gray matter and lower in the white matter [11,12]. In baboon neonates, DHA and AA have been found to be present at highest concentrations in the precentral, postcentral, prefrontal and occipital cortices, and also in basal ganglia, hippocampus, thalamus and cerebellum [12]. As these areas are mainly involved in the development of sensor-motor integration, attention and memory functions, these data suggest the critical role of LC-PUFA in promoting these skills.

Experimental studies confirmed that a deficient dietary intake of DHA can lead to a reduced neuronal cell size, a decreased visual function and a compromised learning behavior [13,14].

DHA and AA can be directly provided from the diet or be synthesized from endogenous conversion of the precursors alpha-linolenic acid (ALA; 18:3ω3) and linoleic acid (LA; 18:2ω6) by enzymatic processes, which are present also in fetuses and infants. However, these enzymatic systems seem to be unable to satisfy LC-PUFA requirements in infants until 16 weeks after birth [15,16]. Accordingly, LC-PUFA during pregnancy are mainly supplied by the placental transfer, whereas during the first months of life the infant is dependent on LC-PUFA supply through breast milk or formula [17].

Fatty acid status can be evaluated by measuring the fatty acid composition of functional tissue such as brain or retina. However, in clinical studies, substitute parameters can be used. It has been demonstrated that erythrocyte membrane fatty acids are representative of brain cell membranes composition, whereas serum lipid levels are influenced by other transient factors, such as contingent diet [18,19]. Therefore, DHA levels in erythrocyte membrane phospholipids are commonly used as an indicator of brain DHA status.

When breastfeeding is not possible, milk substitutes represent the nutritional alternative. It has been demonstrated that formula fed infants have lower levels of LC-PUFA in their cerebral cortex than breastfed infants. This relative deficit of LC-PUFA may partially explain the lower Intelligence Quotient scores reported in formula fed infants in comparison with breastfed infants [20,21]. Makrides M. et al. [22] reported a positive correlation between the erythrocyte DHA levels and the visual-evoked potential acuity. In addition, the authors demonstrated that full-term formula fed infants had erythrocyte DHA levels lower than breast fed infants [22]. Farquharson J. et al. [23] have found lower levels of LC-PUFA in the cerebral cortex of infants fed a formula enriched only with EFA than in breastfed infants. These results suggest that formulas containing only ALA and LA may not be adequate to satisfy the actual requirements of infants in terms of LC-PUFA. Furthermore, it has been reported that infants fed a formula supplemented with DHA and AA have higher erythrocyte membrane omega-3 concentrations at 9 months of age as compared to infants fed an unsupplemented formula [24]. Indeed, supplementation of infant formula with LC-PUFA appears to be associated with a beneficial effect on short-term neurodevelopmental outcome and visual function [25-27]. Although evidence concerning the persistence of the beneficial effect beyond the fourth month of age is lacking, it cannot be excluded that the positive effect of LC-PUFA supplementation may become again evident at school age when infants are required to perform tasks that necessitate more complex neural functions [15].

Besides, DHA consumption appears to influence infants’ body composition by promoting the development of fat free mass without any detrimental effect on growth [27]. Courville AB. et al. [28] have recently demonstrated that infants of mothers consuming food supplemented with DHA during the last half of pregnancy have lower ponderal indices and umbilical cord blood insulin concentrations than infants of mothers consuming the placebo.

Most of infant formulas available nowadays on the market contain plant oils as the only source of fat [29]. Indeed, infant formulas have been enriched in EFA-rich plant oils as cow milk fat does not contain enough EFA to meet infant’s needs [30]. The main plant oils used are coconut oil, corn oil, soybean oil, palm olein, palm kernel oil, palm oil, high oleic safflower oil, peanut oil, and, in Europe, low-erucic acid rapeseed oil. Vegetable oil-based formulas can contain up to 4% residual milk fat [31]. However, plant oils do not contain specific fatty acids, particularly short chain fatty acids, that are present in human and cow milk [32,33] and constitute a pertinent energy source for infants [31]. In addition, plant oils do not encounter milk fat triglyceride structure [30].

The supplementation of infant formulas with dairy lipids could provide a fat composition and structure closer to human milk, thus improving the quality of formula fat composition. Dabadie et al. [34] demonstrated that dairy lipids associated with rapeseed oil significantly increased erythrocyte DHA levels adults. Recently, it has been reported that rodents consuming a diet with a mix of dairy lipids and plant oils showed levels of brain DHA higher than rodents consuming a diet containing only plant oils or a DHA-enriched diet containing plant oils, with the same ALA contents [35]. These data suggest that a mix of dairy lipids and ALA-rich plant oils could potentiate endogenous n-3 LC-PUFA synthesis.

### Study aims

#### Primary aim

To investigate the effect of an infant formula supplemented with a mixture of dairy lipids and plant oils (formula A) on the erythrocyte membrane omega-3 fatty acid profile in healthy full-term infants as compared to a formula containing only vegetable lipids (formula B) or vegetable lipids supplemented with LC-PUFA (AA + DHA) (formula C).

#### Secondary aims

1) To compare erythrocyte membrane LC-PUFA content of infants consuming formula A in comparison to breastfed infants (reference group).

2) To compare the changes throughout the study in blood fatty acids content exhibited by infants consuming formula A in comparison to infants consuming formula B and formula C and to breastfed infants (reference group).

3) To compare the plasma lipid profile and the insulin-growth factor 1 (IGF-1) levels exhibited by infants consuming formula A in comparison to infants consuming formula B and formula C and to breastfed infants (reference group).

4) To investigate the gastrointestinal tolerance of formula A.

5) To evaluate the growth and the body composition changes exhibited by infants consuming formula A in comparison to infants consuming formula B and formula C and to breastfed infants (reference group).

6) To compare the erythrocyte membrane fatty acid profile exhibited by infants consuming formula A in comparison to infants consuming formula B and formula C and to breastfed infants (reference group).

## Methods and design

The study is designed as a mono-centric, double-blind, randomized controlled trial. The Ethical Committee of the Fondazione IRCCS Cà Granda Ospedale Maggiore Policlinico, University of Milan, Italy, approves the study protocol before the start of the study. Written informed consent is obtained from both parents.

### Study population

All infants who are born in the Department of Neonatology of the Fondazione IRCCS Cà Granda Ospedale Maggiore Policlinico, University of Milan, are screened for participation in the study.

Inclusion criteria are: gestational age 37 to 42 weeks, birth weight >2500 g, healthy newborns from normal pregnancy, aged up to 3 weeks when entering the study. Exclusion criteria are: newborns whose parents have planned to move within 6 months after birth, newborns with a positive family history of allergy to milk proteins, newborns with known congenital or postnatal diseases which could interfere with the study. With regard to the reference group, additional inclusion criteria were the intention of mothers to exclusively breastfeed their infants at least for 4 months and being exclusively breastfed at time of enrollment.

### Subject enrollment and randomization

Mothers are encouraged to breastfeed their newborn for at least 4 months. Only if mothers cannot or intend not to breastfeed their newborns, the study team asks the parents for their consent to participate in the study and to be randomized to one of the three formula groups. Enrollment and randomization occur at the same time and are performed within 21 days after delivery.

Infants whose mothers indicate that they intend to exclusively breastfeed are included in the breastfeeding group. This group serves as the non-randomized reference group.

The randomization schedule (provided by Lactalis Recherche et Developpement, Nutrition Department, Retiers, France) is computer-generated and stratified on sex. Sequentially numbered tins of infant formula are prepared according to this schedule. All formulas are packaged in blinded containers labeled only with the study details and the number of randomization; they are indistinguishable in appearance and texture. Once the newborn is enrolled, he/she is allocated to the next available study number which corresponds to the allocation to one of the 3 study formulas. Both the investigators and the infants’ parents are blind to the group allocation.

### Interventions

At enrollment, each newborn is allocated to one of the three formula groups. Newborns randomized to formula A receive a formula containing a mixture of dairy lipids and plant oils; newborns randomized to formula B receive a formula containing only plant oils; newborns randomized to formula C receive a formula containing plant oils supplemented with AA and DHA. Compositions of the three formulas are detailed in Table 1. Study formulas are started straightaway after randomization and are provided for the four subsequent months. The study formulas are formulated into powders and are reconstituted at 13.3%. All study formulas are currently on the market; they are manufactured and provided by Lactalis, Craon, France in compliance with the European directive on infant formulae.

**Table 1:** Composition of the infant formulas

		**Formula A**	**Formula B**	**Formula C**
		100 ml*	100 ml*	100 ml*
Energy	kJ	275	275	275
	kcal	66	66	66
Protein	G	1.3	1.3	1.3
Carbohydrates	G	8.1	8.1	8.1
Lactose	G	6.8	6.8	6.8
Fat	G	3.1	3.1	3.1
Linoleic acid	mg	439	549	549
alpha linolenic acid	mg	73	55	55
AA	mg			12.4
DHA	mg			6.2

* Reconstituted 13.3%.

Infants are fed on demand and are not allowed to introduce complementary foods while receiving one of the study formulas.

### Study outcome measures

The study schedule is reported in Table 2.

**Table 2:** Study schedule

	**V1**	**V2**	**V3**	**V4**	**V5**
Written informed consent	x				
Personal and family history	x				
Inclusion and exclusion criteria	x				
Randomization	x				
Phone calls		x		x	
Heel stick blood sampling	x				x
Venipuncture					x
Anthropometric parameters (weight, length, head circumference)	x		x		x
Body composition	x		x		x
Evaluation of onset and severity of atopic dermatitis by means of the SCORAD index	x		x		x
Adverse events	Throughout the study				

V1=enrollment ; V2= consumption of the allocated formula for one month; V3= consumption of the allocated formula for two months; V4= consumption of the allocated formula for three months; V5= consumption of the allocated formula for four months.

#### Measurement of erythrocyte membrane and blood fatty acid levels, plasma lipid profile (triglycerides, high density lipoprotein, low density lipoprotein, total cholesterol) and IGF-1 levels

Venous blood samples are drawn after consumption of the allocated formula for 4 months. The blood is collected on heparin. Plasma is separated by 15-min centrifugation (2200 g at 4°C) from erythrocytes that are rinsed with saline solution (NaCl 0.9%). The plasma and erythrocytes are stored at −80°C for later analysis.

Erythrocyte membrane FA levels are measured by means of a gas–liquid chromatography (HPLC) by ITERG, Pessac, France. Sum of omega-3 FA levels includes ALA, EPA (eicosapentaenoic acid, 20:5n-3), DPA (docosapentaenoic acid, 22:5n-3) and DHA levels. Plasma lipid profile and IGF1 levels are measured by means of standard techniques.

#### Measurement of blood FA levels

Whole blood samples are collected using a heel stick at enrollment and after four months of consumption of the allocated formula. FA levels are quantified by means of a gas–liquid chromatography (HPLC) by Oxigenlab, Brescia, Italy.

#### Investigation of the gastrointestinal tolerance

Parents are contacted every four weeks either by clinic visits or phone calls. Specifically, the following indicators of gastrointestinal tolerance are collected after one and three months of consumption of the allocated formula: amount of formula consumed, regurgitation/reflux, colic (intermittent attacks of abdominal pain when the baby screams and draws up his/her legs but is well between episodes), daily frequency of stool passage, stool consistency and color. In addition, during the clinic visits, the onset, if present, and the severity of atopic dermatitis is evaluated by means of the SCORAD index [36].

#### Growth and body composition

Birth weight, length and head circumference are recorded. Growth and body composition are then evaluated at enrollment, after 2 and 4 months of consumption of the allocated formula. The infants’ anthropometric measurements (body weight, length, head circumference) are obtained using standardized techniques [37]. Subject mass is measured on an electronic scale accurate to the nearest 0.1 g. Recumbent length is measured on a Harpenden stadiometer to the nearest 1 mm. The head circumference is measured using a non-stretch measuring tape to the nearest 1 mm. Z-scores for weight, length and head circumference are then calculated with the EUROGROWTH software (http://www.euro-growth.org/). Body composition is assessed using an air displacement plethysmography system (PEA POD Infant Body Composition System, COSMED- USA). A detailed description of the PEA POD’s physical design, operating principles, validation and measurement procedures is provided elsewhere [38,39]. The PEA POD assesses fat mass and fat free mass by direct measurements of body mass and volume and the application of classic densitometric principles. Infants are measured in the PEA POD naked. Each PEA POD test takes about 3 min to complete. Subject volume is measured in an enclosed chamber by applying gas laws that relate pressure changes to volumes of air in the chamber. Body density is then computed from the measured body mass and volume, and inserted into a standard formula for estimating the percentage of total body fat mass according to a 2- compartment model. The intra-observer coefficient of variation for the percentage of fat mass estimates is 0.3%.

### Sample size, power and statistical analysis

In order to detect a difference of 20% with a standard deviation of 0.3 in the erythrocyte membrane omega-3 fatty acids levels between infants receiving formula A and infants receiving formula B or formula C, at a power of 90% and with an alpha error of 5%, a total of 23 infants per group is necessary. Considering a drop out rate of about 10%, a total of 75 formula fed infants, with 25 infants for each group, are included. Twenty-five exclusively breastfed infants constitute the reference group.

All statistical analyses are performed on an intention to treat basis. In addition, alternative per protocol analyses are performed, excluding all infants who are not fed according to the protocol study. Statistical analysis is performed by Soladis, Lyon, France. For statistical analysis a p-value <0.05 is considered significant (two tailed). All statistical analyses are performed using SPSS 12 (SPSS Inc., Chicago, IL, USA). Continuous variables are expressed as mean, standard deviation, minimum, maximum, median, quartiles.

The differences among groups in erythrocyte omega-3 FA levels, plasma lipid profile and IGF-1 levels are assessed using an analysis of covariance with sex as covariate. Differences among and within groups in repeated measurements of plasma FA levels, of growth parameters and body composition, are analyzed with an analysis of variance.

As the breastfed infants are not randomized, no statistical analysis is performed to compare the breastfed group with any of the formula feeding groups.

### Adverse events and serious adverse events

Adverse events (AEs) are assessed based on clinical observation of the investigators or inquires to the parents. All AEs are recorded in adverse event forms and are evaluated by the investigator for causality for the relationship to the study feeding and for severity. An AE is defined as any event, that is not consistent with the information provided in the consent form or can reasonably be expected to accompany the natural history and progression of the subject’s condition throughout the study. AEs are considered as serious (SAEs) if they are fatal or life-threatening, require hospitalization or surgical intervention, result in persistent or significant disability/incapacity or are considered to be medically relevant by the investigator. All other AEs are categorized as non-serious.

## Discussion

Human milk represents the optimal nutrition for infants and ensures optimal growth and development. LC-PUFA, which are provided through breast milk, play a critical role in the development of neuronal and visual functions. Hence, the achievement of an appropriate neonatal LC-PUFA status represents an important goal in neonatal nutrition, especially when breastfeeding is not possible [5,7,8]. Formula fed infants have been demonstrated to exhibit lower cerebral and erythrocyte LC-PUFA levels as compared to breast fed infants when consuming an unsupplemented formula or a formula supplemented only with EFA [22,23]. On the contrary, infants receiving a formula supplemented with DHA and AA show higher erythrocyte membrane omega-3 concentrations than infants fed an unsupplemented formula [24]. Recent experimental data indicate that a dairy fat blend providing as little as 1.5% ALA is superior to the plant oil blend for increasing brain DHA, even when the recommended DHA and AA levels are exogenously provided, suggesting that the endogenous synthesis of n-3 LC-PUFA synthesis can be enhanced [35]. Formula fed infants could then benefit from the supplementation of formula with dairy lipids and plant oils by attaining higher erythrocyte membrane omega-3 FA content than infants receiving a formula supplemented with plant oils or plants oils enriched with LC-PUFA. The achievement of optimal DHA levels in formula fed infants could also be associated with improved neurocognitive and visual functions, as previously underlined by other studies [25-27]. Not only the erythrocyte membrane omega-3 FA levels will be investigated but also the other erythrocyte membrane FA levels, the plasma lipid profile, the insulin growth factor 1, the formula tolerance, the growth and body composition. Gaining insight in the effects of the supplementation of a formula with dairy lipids and plant oils in healthy full-term infants could improve the fat content, composition and structure of infant formulas, making them closer to human milk.

## Abbreviations

AE: Adverse event; ALA: Alpha linolenic acid; AA: Arachidonic acid; DHA: Docosahexaenoic acid; DPA: Docosapentaenoic acid; EFA: Essential fatty acid; EPA: Eicosapentaenoic acid; FA: Fatty acid; IGF1: Insulin-like growth factor 1; LA: Linoleic acid; LC-PUFA: Long chain polyunsaturated fatty acid; SAE: Serious adverse event.

## Competing interests

Lactalis Nutrition Santé, Torcé, France for the financial support and for providing the study formulas.

## Authors’ contributions

Paola Roggero, Pascale Le Ruyet and Fabio Mosca formulated the research question and wrote the study protocol. Daniela Morniroli, Amandine Ligneul contributed to the development of the protocol. Francesca Garbarino gave advice on statistical analysis. Charlotte Baudry, Maria Lorella Giannì, Paola Roggero wrote the draft of the manuscript and the other authors reviewed the manuscript. All authors approved the final version of the manuscript.

## Pre-publication history

The pre-publication history for this paper can be accessed here:

http://www.biomedcentral.com/1471-2431/12/164/prepub
